# Microwave-Assisted Catalytic Deconstruction of Plastics Waste into Nanostructured Carbon and Hydrogen Fuel Using Composite Magnetic Ferrite Catalysts

**DOI:** 10.1155/2024/3318047

**Published:** 2024-05-31

**Authors:** Bilal Shoukat, Hammad Hussain, Muhammad Yasin Naz, Ahmed Ahmed Ibrahim, Shazia Shukrullah, Yasin Khan, Yaning Zhang

**Affiliations:** ^1^Department of Physics, University of Agriculture Faisalabad, Faisalabad 38040, Pakistan; ^2^Department of Agricultural Engineering, Faculty of Agricultural Engineering & Technology, University of Agriculture Faisalabad, Faisalabad 38040, Pakistan; ^3^Department of Physics and Astronomy, College of Science, King Saud University, P.O. Box 2455, 11451 Riyadh, Saudi Arabia; ^4^Department of Electrical Engineering, College of Engineering, King Saud University, Riyadh, Saudi Arabia; ^5^School of Energy Science and Engineering, Harbin Institute of Technology (HIT), Harbin 150001, China

## Abstract

Finding new catalysts and pyrolysis technologies for efficiently recycling wasted plastics into fuels and structured solid materials of high selectivity is the need of time. Catalytic pyrolysis is a thermochemical process that cracks the feedstock in an inert gas environment into gaseous and liquid fuels and a residue. This study is conducted on microwave-assisted catalytic recycling of wasted plastics into nanostructured carbon and hydrogen fuel using composite magnetic ferrite catalysts. The composite ferrite catalysts, namely, NiZnFe_2_O_4_, NiMgFe_2_O_4_, and MgZnFe_2_O_4_ were produced through the coprecipitation method and characterized for onward use in the microwave-assisted valorization of wasted plastics. The ferrite nanoparticles worked as a catalyst and heat susceptor for uniformly distributed energy transfer from microwaves to the feedstock at a moderate temperature of 450°C. The type of catalyst and the working parameters significantly impacted the process efficiency, gas yield, and structural properties of the carbonaceous residue. The tested process took 2–8 minutes to pulverize feedstock into gas and carbon nanotubes (CNTs), depending on the catalyst type. The NiZnFe_2_O_4_-catalyzed process produced CNTs with good structural properties and fewer impurities compared to other catalysts. The NiMgFe_2_O_4_ catalyst performed better in terms of hydrogen evolution by showing 87.5% hydrogen (H_2_) composition in the evolved gases. Almost 90% of extractable hydrogen from the feedstock evolved during the first 2 minutes of the reaction.

## 1. Introduction

Plastic manufacturing has increased twentyfold over the past fifty years, which is predicted to continue in the coming years. By 2050, the yearly output of plastics will approach 500 million tons, using up as much as 40% of the world's crude oil [1]. More than 80% of plastic ever manufactured is finished in the environment due to disposal of plastic materials in landfills and marine and coastal environments. Currently, at most, 2% of plastic production comes from forming renewable resources. Furthermore, only 14% of plastic waste is recycled and only 2% of that material is recovered for use in applications that require the same or equivalent quality materials. Currently, mechanical recycling is the primary process used to recycle plastics, and this method usually entails collecting, sorting, and cleaning the material before processing it to create new products [2]. As the need for plastic in the modern world grows, so does the amount of waste plastic. The two most popular methods for treating waste plastics produced by the municipal solid waste stream are landfilling and incineration [3]. The energy content of used plastics is lost when disposed of in landfills. Although energy is recovered during incineration, the carbon content of the plastics is primarily converted to carbon dioxide (CO_2_) and released into the atmosphere. The thermal cracking of plastic waste via pyrolysis is among the top favorable chemical recycling methods.

Plastic is thermally decomposed during pyrolysis into a liquid like oil or wax and small quantities of gaseous products or solid residue leftovers [4]. The pyrolysis of waste plastic for H_2_ production has been abundantly researched. Various catalysts have also been researched to make the procedure efficient and increase hydrogen production [5]. More work on catalyst development is needed to improve the yield and structural properties of the solid residue for its practical use as a nanomaterial. In the present research, the role of ferrite catalysts was determined in producing CNTs as a solid residue and hydrogen gas from the wasted plastics [6]. Ferrite nanoparticles play a dual role in microwaves energized catalytic pyrolysis of plastic waste. Almost all plastics are thermal insulators and do not absorb heat energy directly from the incident microwaves. Ferrite nanoparticles work as heat susceptors for absorbing energy from microwaves and transferring it to plastic molecules [7]. Concurrently, they provide abundant active sites for redox reactions and nucleation of CNTs. Ferrite nanoparticles have redox capabilities, which means they can easily switch between different oxidation states. This redox behavior benefits CNTs growth because it allows for the activation and regeneration of catalytic sites during the synthesis process. The plastic molecules decompose into carbon, hydrogen, and other gases and byproducts on receiving energy from ferrite nanoparticles. The carbon atoms get adsorbed onto the catalyst surface and then diffuse and dissolve into the catalyst nanoparticles [8]. The high thermal stability, surface area, and chemical reactivity of the magnetic catalyst facilitate the localized heating in this process. The dissolved carbon atoms begin to form nanotube structures. High thermal stability is important for maintaining catalytic activity throughout the growth of CNTs. Conventional pyrolysis often produces tar and char residue, which can interfere with the efficiency of the process. Ferrite nanoparticles can aid in the decomposition and cracking of tar and char, preventing their accumulation and improving the conversion of plastic into valuable products. The catalytic properties of ferrite nanoparticles can enhance the cracking of long-chain hydrocarbons, reducing the formation of undesired byproducts. Magnetic ferrite catalysts are more effective in producing hydrogen due to their high ability to break C-C bonding in the plastic chain and low cost.

Microwaves can permeate solid materials to commence volumetric heating, as opposed to traditional heating, which can only achieve surface heating. In microwave pyrolysis of plastics, an absorbent dielectric substance is often used to induce heat transfer to the plastic feedstock. Magnetic catalysts like NiZnFe_2_O_4_, NiMgFe_2_O_4_, and MgZnFe_2_O_4_ absorb more microwave energy than other materials because of their high dielectric loss factor [9]. These catalysts enhance the quality of the process leading to better selectivity of the products even at moderate temperatures compared to conventional pyrolysis. This catalyst effectively minimizes the formation of tar and char by breaking organic molecules. Microwave interactions with these catalysts result in a high Snoek's limit, complex permeability, and saturation magnetization. Many researchers have indicated that microwave-assisted heating is a viable method for the pyrolysis of various feedstocks, particularly polymers [10, 11]. Microwave-catalytic pyrolysis is a complicated thermochemical process that is regulated by various factors such as process temperature, microwave absorbents, and catalyst types [12]. Microwave pyrolysis is gaining popularity due to significant advantages over traditional heating processes, such as contactless volumetric heating, simple heat distribution, fast processing, better energy efficiency, and high pyrolytic oil or combustible gas yield. Zhou et al. [13] assumed that temperature is an important parameter in defining product distribution and energy utilization by the pyrolysis of plastic waste. Microwave heating does not heat the entire sample due to a small temperature differential from the outside surface, which could result in multiple side reactions and the formation of unnecessary byproducts. However, waste plastics absorb low microwave heat energy because they are microwave transparent. Combined with microwave absorbers, such as carbon compounds and inorganic oxide, plastic can easily be degraded. It is also been observed that microwaves strongly interact with magnetic-based catalysts [14]. More microwave energy can be absorbed by coupling ferromagnetic species with the magnetic components of the microwave field. At lower temperatures, the reaction may occur by multiple mechanisms because of continuous heating mechanisms from conventional heating techniques [15]. CNTs and hydrogen are described as the major carbonaceous products other than CO_2_, methane (CH_4_), ethylene (C_2_H_4_), carbon monoxide (CO), etc., during the pyrolysis of plastics. H_2_ can be utilized as an efficient energy source due to high energy density (120.7 kJ/g) and ecofriendly chemistry [16].

The exact mechanism of CNT nucleation during such recycling processes is not fully understood; however, it is widely assumed to involve the formation of atomic carbon, its absorption onto the catalyst surface, and then diffusion and precipitation onto nanotube structures. One notable advantage of ferrite nanoparticles is their magnetic nature. The magnetic properties of ferrite nanoparticles can be exploited to manipulate and control the alignment and orientation of CNTs during growth. Externally applied fields can also influence the growth direction and alignment of CNTs on the catalyst surface. For microwave pyrolysis applications, catalysts with a high dielectric loss factor are the best choice [17]. When exposed to microwave radiation, ferrite nanoparticles experience a phenomenon known as dielectric heating. The electromagnetic field of microwaves interacts with the electric dipoles in the ferrite nanoparticles to cause dielectric heating [18]. This interaction causes the ferrite to rapidly alternate its magnetic polarity, resulting in energy absorption and heat generation. This work reports a microwave-assisted catalytic pyrolysis approach for achieving high hydrogen yield and structured CNTs by recycling high-density polyethylene (HDPE) using composite ferrite catalytic, such as NiZnFe_2_O_4_, NiMgFe_2_O_4_, and MgZnFe_2_O_4_.

## 2. Materials and Methods

### 2.1. Materials

HDPE is an excellent feedstock for hydrogen due to its high hydrogen concentration and abundant availability in municipal waste. The HDPE waste was used in the presented study. The plastic feedstock was sampled from municipal waste materials. The sampled plastic was washed with deionized water to eliminate harmful substances. The plastic was then dried and chopped into 3–5 mm pieces. Waste plastics have a limited fixed carbon and ash percentage and a high volatile matter concentration (around 95.5 wt.%). In addition, higher hydrogen and carbon concentrations have been detected in HDPE wastes. To be more exact, HDPE had a higher carbon content (>70 wt.%) and a higher H_2_ concentration (85 vol.%). Three magnetic catalysts, namely, NiZnFe_2_O_4_, NiMgFe_2_O_4_, and MgZnFe_2_O_4_ were tested for microwave-driven pyrolysis of the prepared feedstock. The exceptional microwave-absorbing property and low cost of magnetic catalysts make them appropriate for use as both microwave susceptors and catalyst support.

### 2.2. Synthesis of Ferrite Catalysts

The nanoparticles of NiZnFe_2_O_4_, NiMgFe_2_O_4_, and MgZnFe_2_O_4_ were synthesized by a coprecipitation process. Iron nitrate (Fe(NO_3_)_3_9H_2_O), magnesium (II) nitrate (Mg(NO_3_)_2_6H_2_O), zinc (II) nitrate (Zn(NO_3_)_2_6H_2_O), and nickel (II) nitrate (Ni(NO_3_)_2_6H_2_O) were used in the same ratio to produce the composite ferrite catalysts. The zinc nitrate, magnesium nitrates, and iron nitrate dissolved into 50 ml of deionized (DI) water. The solution was continuously stirred at 50°C for almost 4 hours to make a completely homogenous mixture [19]. Subsequently, NaOH solution was added dropwise to the mixture to maintain a pH of 12. Dark gray precipitates were produced during this process. After cooling the mixture, the precipitated content washed multiple times with DI water to eliminate contaminants. The solid residue was dried at 90°C for 12 hours. The dried residue was further annealed at 600°C for 8 hours to obtain the final product of MgZnFe_2_O_4_. The same procedure was adopted to prepare NiMgFe_2_O_4_ and NiZnFe_2_O_4_ catalysts [20]. The surface topology and size of the catalyst samples were analyzed by scanning electron microscopy (SEM). The crystal structure of the catalyst samples was probed by using the X-ray diffraction (XRD) spectroscopy method. The optical traits of the synthesized samples were checked by producing UV-vis spectra. Fourier transform infrared (FTIR) spectra of the catalysts were taken at room temperature between 500 and 4500 cm^−1^ for the characterization of functional groups and bond formation.

### 2.3. Pyrolysis Experiments

**Figure 1:**
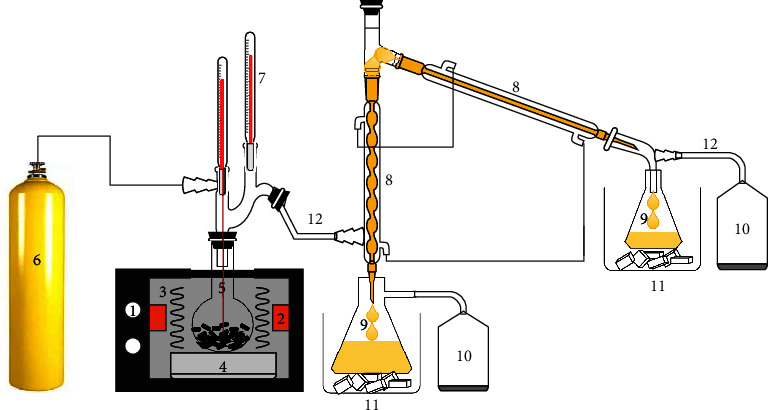
The setup used for pyrolysis of HDPE consists of (1) a microwave source, (2) waveguide, (3) microwave interaction with feedstock, (4) ceramic platform, (5) feedstock container, (6) nitrogen supply, (7) temperature sensor, (8) condenser, (9) oil container, (10) gas sampler (11) cold trap, and (12) glass tubing.

The reported catalytic pyrolysis involved the catalytic decomposition of plastic at 450°C in an oxygen-free chamber. Pyrolysis is the most reliable and straightforward process for recycling plastic waste into oil, H_2_ gas, and valuable solid products. But the end products from this process need to be chemically improved and further purified. The pyrolysis process incorporates an appropriate catalyst to overcome these challenges.  Figure 1 illustrates the experimental setup used in the present study. The catalytic microwave pyrolysis of plastic was conducted in a multimode microwave reactor operated with a 2450 MHz source [21]. The experimental setup contains a gas supply system, a microwave source, a temperature sensors, connecting tubes, condensers, an oil container, cold traps, gas analyzer, and a cold-water supply. A round-bottomed flask was used as a feedstock container, which is transparent to the incident microwaves. To remove any residual air, the pyrolysis chamber was purged with nitrogen gas at a flowrate of 200 ml min^−1^ for 20 minutes just before starting the microwave pyrolysis process [22]. The microwave reactor then operated for 8 minutes at 1000 W power. A mass flow controller was used to control the gas flowrate. The flowrate and composition of the produced gases was measured with a gas analyzer. The gas product was estimated using the obtained mass relative to the total weight of feedstock (equation (1)). The yield of H_2_ is calculated by dividing the moles of H_2_ by the total mass of feedstock (equation (2)). The efficiency of hydrogen production is calculated by dividing the theoretical hydrogen mass in plastic by the total hydrogen mass in all gas products (equation (3)).

(1) $$\begin{array}{c} \text{Gas Yield }\left(\text{wt}.\%\right)=\frac{m_{g}}{m_{p}}\times 100\%. \end{array}$$

(2) $$\begin{array}{c} \text{Hydrogen Yield }\left(\text{mmol}/g_{P}\right)=\frac{m_{H_{2}}}{m_{P}}. \end{array}$$

(3) $$\begin{array}{c} \text{Hydrogen Efficeincy}\left(\%\right)=\frac{m_{g_{H}}}{m_{Th}}, \end{array}$$

where *m*_*g*_ is the mass of gas, *m*_*p*_ is the plastic waste,*m*_*H*_2__ is the moles of H_2_, m_th_ is the theoretical H_2_ mass in plastic waste, and m_gH_ is the total H_2_ mass present in all gaseous products [23]. The yield of the produced gas can be determined using equations (1)–(3).

For pyrolysis experiments, 40 g of feedstock and 10 g of the catalyst were mixed uniformly in a weight ratio of 4 : 1. The feedstock was placed in the microwave oven in a round-bottomed quartz flask [24]. Three sets of experiments were conducted using NiZnFe_2_O_4_, NiMgFe_2_O_4_, and MgZnFe_2_O_4_ at 450°C [25]. The catalyst plays an essential role in the reaction because it produces the desired products at a low temperature. Magnetic ferrite catalysts catalyze the reaction and work as microwave heat susceptors. Temperature sensors were added to the setup to monitor the feedstock and gas outlet temperature. The feedstock temperature was maintained at 450°C throughout the pyrolysis process. Liquid oil was condensed and collected using a one-necked flask and a cooling column was cooled using chilled water. The noncondensable gas was analyzed using a gas analyzer [26].

### 2.4. Characterization of the Product

SEM and XRD techniques were employed to characterize the fresh catalysts and the solid products of the pyrolysis of plastic. The parameters of the products, such as structure, grain size, lattice parameter, and unit cell volume, were studied by producing XRD patterns. The crystallinity was evaluated from the ratio of the integrated peak area for the crystalline phase and amorphous fraction area. The average crystallite size was determined using the Scherrer equation. SEM was used to analyze morphology and to identify the clusters and pores on the product surface. Thermogravimetric analysis (TGA) was performed to check thermal stability of the samples. FTIR spectra of the pyrolysis products and catalysts were produced at room temperature between 500 cm^−1^ and 4500 cm^−1^ to characterize functional groups and the bond formation of the samples. The gas analyzer was utilized to assess the composition of the evolved gas.

## 3. Results and Discussion

### 3.1. Structural Characteristics and Optical Response of Catalysts

**Figure 2:**
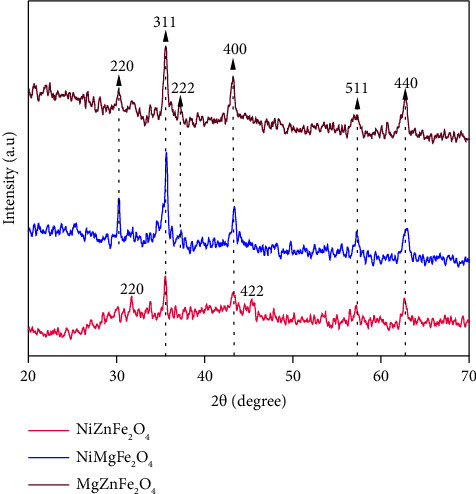
XRD patterns of NiZnFe_2_O_4_, NiMgFe_2_O_4,_ and MgZnFe_2_O_4_ catalysts.

The crystalline solid structure of the prepared catalysts was analyzed using the most prominent diffraction peaks in the XRD patterns. Peak variations have been used to determine and explain multiple phases and specifications of the produced ferrite catalysts [27]. X-ray diffractograms of NiZnFe_2_O_4,_ NiMgFe_2_O_4,_ and MgZnFe_2_O_4_ catalysts are reported in Figure 2. The diffraction peaks of all samples are within 2*θ* range of 20°–70°. The XRD peaks of the cubical spinel structure of NiZnFe_2_O_4_ mostly exist at 2*θ* of 31.20°, 35.58°, 43.24°, 44.31°, 57.17°, and 62.65°, corresponding to (220), (311), (400), (422), (511), and (440) crystal planes, which match the basic NiZnFe_2_O_4_ JCPDS card no. 52-0278. The observed peaks of NiMgFe_2_O_4_ and MgZnFe_2_O_4_ mostly exist at 2*θ* of 30.20°, 35.58°, 37.16°, 43.24°, 57.17°, and 62.65°, corresponding to (220), (311), (222), (440), (511), and (440) crystal planes, which match the basic NiMgFe_2_O_4_ JCPDS card no. 08-0234 and MgZnFe_2_O_4_ JCPDS card no. 22-1086 corresponding to (311) peak [28].

A prominent peak for (311) phase was selected to calculate the grain size of the catalyst nanoparticles [29]. The prepared samples showed the cubical spinel phase structure and Fd-3m space group. Debye-Scherrer's equation was considered to estimate the crystallite size as

(4) $$\begin{array}{c} D=\frac{0.9\lambda }{\beta \mathrm{Cos}\theta }, \end{array}$$

where *λ* is wavelength of X-ray (1.5406 Å), *θ* is the diffraction angle, *β* represents the FWHM of the XRD peak, and 0.9 is the Scherrer's constant [30]. The average grain size of NiZnFe_2_O_4,_ NiMgFe_2_O_4,_ and MgZnFe_2_O_4_ is determined as 17, 39, and 35.46 nm, respectively. A summary of the XRD analysis is provided in Table 1.

**Figure 3:**
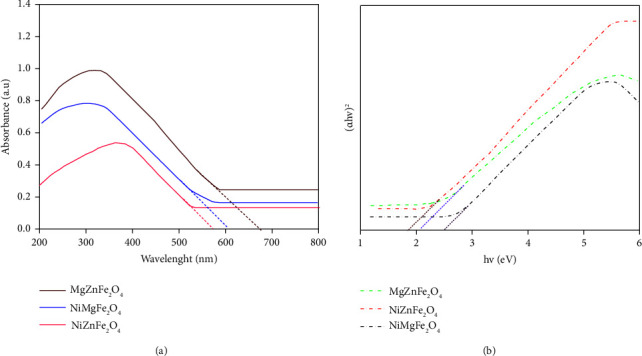
(a) UV-vis analysis of NiZnFe_2_O_4_, NiMgFe_2_O_4,_ and MgZnFe_2_O_4_ and (b) optical band gap of these ferrite catalysts.

The light absorption spectra were produced to determine the optical properties of NiZnFe_2_O_4_, NiMgFe_2_O_4_, and MgZnFe_2_O_4_ ferrite catalysts. The continuous absorption spectra of these samples in 200–800 nm range are shown in Figure 3(a). Maximum absorption was observed between the wavelength range of 570–680 nm, indicating a violet shift in the absorption spectrum [31]. The band gap of these catalyst nanoparticles was calculated using Tauc's relation.

(5) $$\begin{array}{c} {\left(\alpha hv\right)}^{2}=A\left(hv\;‐\;E_{g}\right). \end{array}$$

In equation (5), constant “*A*” depends on the probability of the transition, *α* is the absorption coefficient, “*E*_*g*_” is band gap, “*hv*” is the energy of the incident photon. We calculated the [band gap] of the synthesized nanoparticles as a function of the curve between the optical band gap energy at *X*-axis and the curve between (*αhv*)^2^ and (*αhv*)^1/2^ at *y*-axis using an extrapolation of the curve between the optical band gap energy at *x*-axis. All these graphs are shown in Figure 3(b). Nanoparticles of NiZnFe_2_O_4_, NiMgFe_2_O_4_, and MgZnFe_2_O_4_ have a band gap of 2.1 eV, 2.5 eV, and 1.9 eV, respectively [32].

**Figure 4:**
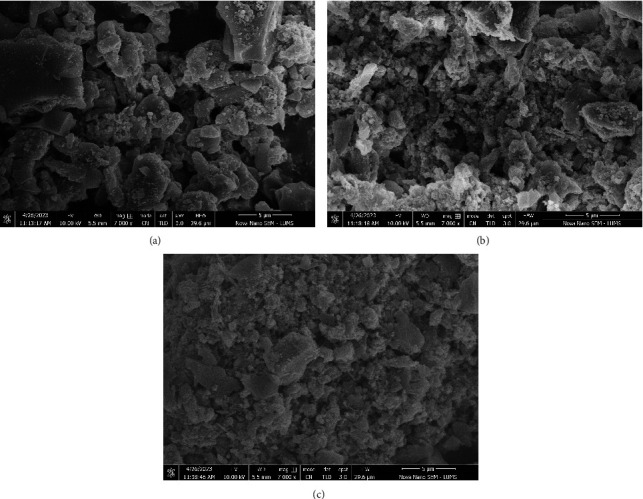
SEM images of (a) NiZnFe_2_O_4_ (b) MgZnFe_2_O_4_, and (c) NiMgFe_2_O_4_ catalysts.

Figure 4 shows the morphology of NiZnFe_2_O_4,_ NiMgFe_2_O_4,_ and MgZnFe_2_O_4_ catalysts. The SEM images revealed the rough morphology of all catalysts having irregular particle shapes and sizes. Some degree of agglomeration was also observed among the catalyst particles. MgZnFe_2_O_4_ showed fine particle size, followed by NiMgFe_2_O_4_ and NiZnFe_2_O_4_ catalysts [33]. Magnetic interaction could be a possible reason for agglomeration among the catalyst particles. NiZnFe_2_O_4_ particles were relatively more dispersed and larger in size with a reduced degree of agglomeration. The high aggregation of nanoparticles may reduce the surface area and thereby density of active sites to perform the catalytic reaction [34].

The active sites also reduce with an increase in the particle size. NiZnFe_2_O_4_ catalyst showed the least aggregation of particles but a large particle size. At the same time, the particle shape and boundaries of this catalyst were more defined than the other catalyst. These characteristics could significantly boost the number of active sites and hereinafter the catalytic activity of the catalysts. Therefore, NiZnFe_2_O_4_ was regarded as a good candidate to demonstrate good catalytic activity to pyrolyze HDPE [35]. The mean particle size of NiZnFe_2_O_4_, NiMgFe_2_O_4_, and MgZnFe_2_O_4_ catalysts was measured to be 64 nm, 51 nm, and 48 nm, respectively [36].

**Figure 5:**
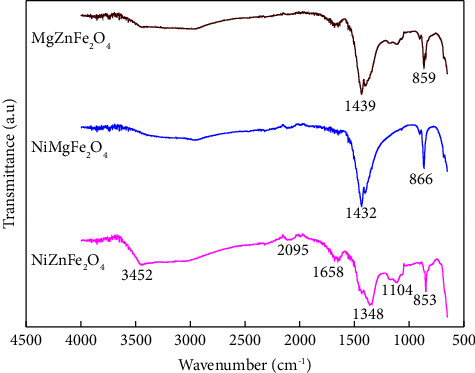
FTIR spectra of NiZnFe_2_O_4_, NiMgFe_2_O_4_, and MgZnFe_2_O_4_ catalysts.

FTIR spectrum represented different transmittance bands in catalysts because of the vibration of functional groups and the vibration of ions at the lattice sites. Due to the vibrations of functional groups, the bands usually appear in 500–4500 cm^−1^ range. The FTIR spectra of NiZnFe_2_O_4,_ NiMgFe_2_O_4_, and MgZnFe_2_O_4_ catalysts are represented in Figure 5. The vibrating modes at ∼3452, 2095, 1658, 1348, 1104, 853, and 654 cm^−1^ demonstrated the functional groups of catalyst samples and the pure phasic spinel formation [37]. The bands at 3452 and 1658 cm^−1^ represent O-H stretching and bending vibrations of water molecules. The bands between 1513 and 1663 cm^−1^ range are caused by symmetric and asymmetric C-O group vibrations [38]. The band around 1348 cm^−1^ represents the existence of moisture. The stretching of C-O bonds is comparable to the vibrating band at 1104 cm^−1^. The bands below 1000 cm^−1^ are attributed to octahedral M-O and tetrahedral stretching vibration modes [39].

### 3.2. Role of Catalyst Properties in Pyrolysis

The structural and morphological traits of a catalyst play a crucial role in influencing the activation energy of the reactions involved in pyrolysis and nucleation of CNTs. The studied magnetic catalysts would increase the reaction rate and make the pyrolysis process more efficient by giving an alternate reaction pathway with lower activation energy. The size and shape of catalyst particles can influence the accessibility of reactant molecules to active sites. Smaller particles may have more surface area and exposed active areas, resulting in lower activation energy. Furthermore, due to differences in electrical and geometric attributes, various surface facets or crystal structures of catalysts can have different catalytic activities. The tested catalysts showed the same electronic and geometric properties but slightly different particle sizes [36]. Although NiZnFe_2_O_4_ catalyst had the least particle aggregation, the largest particle size, shape, and boundaries of the particles were more defined than the other catalysts. These properties could significantly increase the number of active sites and, as a result, the catalytic activity of the catalysts. NiZnFe_2_O_4_ was recognized as a promising candidate for pyrolyzing HDPE with a strong catalytic activity [35]. The active sites on the magnetic catalyst surface aided in the adsorption and activation of reactant molecules, allowing the reaction to proceed more quickly. As a result, the reactions involved had lower activation energy.

The precise process of CNT nucleation during catalytic pyrolysis is unknown. It is widely thought, however, that the process involves the synthesis of atomic carbon, its absorption into the catalyst surface, and subsequently, diffusion and precipitation onto nanotube structures [17]. Reactant adsorption can stabilize the transition state, lowering the energy required to achieve the activated complex and the activation energy. Since all the tested catalysts were redox active, they transfer electrons between the catalyst surface and reactant molecules, facilitating the reaction and reducing the activation energy for the formation of nanotubes. Since magnetic catalysts were composite materials, the iron oxide works in conjunction with nickel (Ni), magnesium (Mg), and zinc (Zn), leading to synergistic effects. The combined action of different catalytic materials can further decrease the activation energy and improve the overall catalytic performance.

### 3.3. Characteristics of the Solid Residue

**Figure 6:**
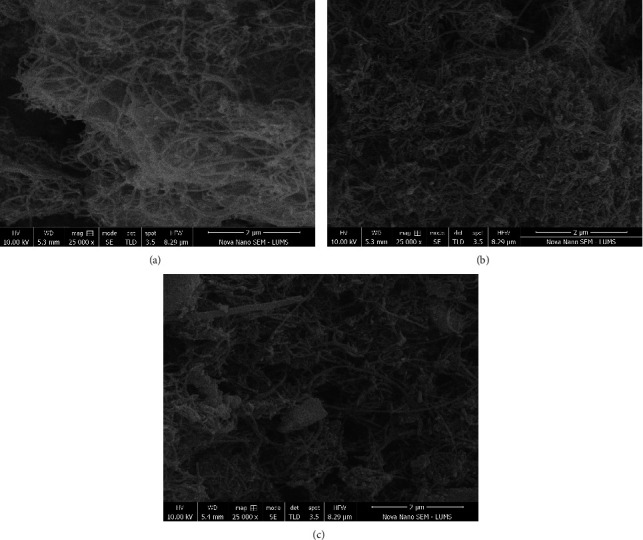
SEM micrographs of pyrolysis residue produced with (a) NiZnFe_2_O_4_, (b) NiMgFe_2_O_4_, and (c) MgZnFe_2_O_4_ catalysts.

The surface morphology of CNTs is displayed in Figure 6. It is observed that CNTs produced using NiZnFe_2_O_4_ as a catalyst revealed threadlike structures with nonuniform diameters and lengths. Most CNTs exhibited clear boundaries and shapes, while some joined to form clustered structures, consistent with earlier documented studies [8, 40]. Such structure formations are assigned to van der Waals forces, entropy-driven agglomeration, and electrostatic interactions between CNTs.  Figure 6(a) shows relatively longer CNTs, while Figure 6(b) displays the formation of CNTs with complex structures in the presence of NiMgFe_2_O_4_ catalyst [41]. Some particles of the ferrites remained rounded, identifying the presence of unutilized catalyst even after the pyrolysis process was complete. The presence of catalyst particles in CNTs can be attributed to strong chemical bonding between the catalyst and CNTs, which reduces the diffusion of the catalyst into the feedstock [42]. It was also witnessed that the CNTs produced in the presence of NiMgFe_2_O_4_ displayed large diameters as compared to other catalysts.  Figure 6(c) revealed the densely clumped surface of CNTs made in the presence of MgZnFe_2_O_4_. Several CNTs structures with different lengths and diameters were produced. The SEM analysis revealed that NiZnFe_2_O_4_ catalyst produced relatively better quality CNTs compared to NiMgFe_2_O_4_ and MgZnFe_2_O_4_, and these findings support the catalyst properties reported in previous sections [43]. These arguments agree with the diameter of the produced CNTs in the presence of different catalysts with different particle sizes [44].

**Figure 7:**
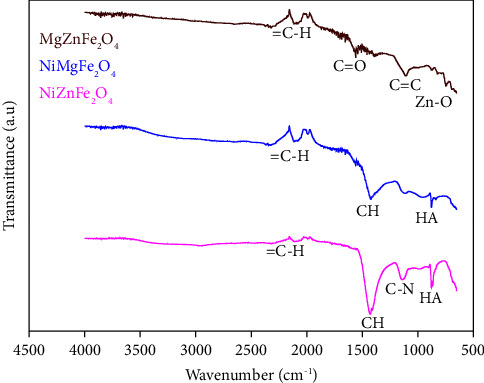
FTIR of solid residue of the pyrolysis process catalyzed with NiZnFe_2_O_4_, NiMgFe_2_O_4_, and MgZnFe_2_O_4_ catalysts.

The functional groups in the compounds were identified based on their absorption frequencies. According to Figure 7, bands were observed at 1089 cm^−1^ corresponding to carboxyl bond oscillations and at 1453 cm^−1^, indicating aromatic ring bonds [45]. FTIR analysis showed the formation of the aromatic benzene group at 1182 cm^−1^ and a significant carbon absorption bond peak observed for =C-H in the range of 2105−2095 cm^−1^ [46]. Other characteristic peaks of CNTs include CH at 1425–1434 cm^−1^ and C-N at 1136 cm^−1^ due to stretching vibration and 1040 cm^−1^ representing the functional groups of CoC of HA. The vibration of C=C can be seen in the range of 748–768 cm^−1^ [47]. Furthermore, C=O bonding vibration in CNTs is recorded between the range of 1575 and 1595 cm^−1^. The peak detected at around 756 cm^−1^ is attributed to tetrahedral site Zn-O stretching, while the band detected at 689 cm^−1^ is attributed to Fe-O vibration at the octahedral sites [48]. The results from this study demonstrated the presence of functional groups as well as spectrum vibrations of the carboxyl group and amide bonds in the product.

**Figure 8:**
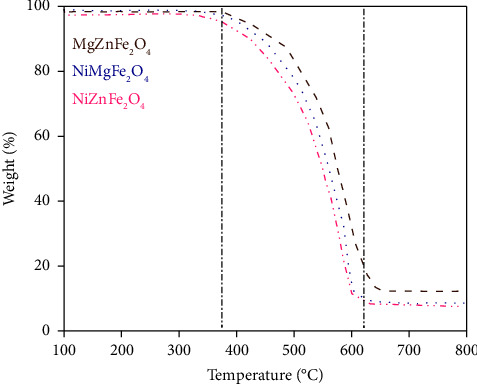
TGA profiles of the solid residue of the pyrolysis catalyzed with NiZnFe_2_O_4_, NiMgFe_2_O_4_, and MgZnFe_2_O_4_ catalysts.

The TGA is depicted in Figure 8, indicating the thermal behavior of the residue of catalytic pyrolysis of plastic. The weight of the samples is plotted at different temperatures to demonstrate thermal shifts in the solid material on the TGA curve. Since the pyrolysis was conducted at 450°C, the residual mass may contain char, unspent catalyst, and other contaminants present in the feedstock composition [49]. TGA analysis demonstrated small quantities of amorphous carbon in the product obtained with NiZnFe_2_O_4_ catalyst. On the other hand, NiMgFe_2_O_4_ and MgZnFe_2_O_4_ showed slightly higher amorphous content in the solid product [50]. The TGA analysis showed 7.8%, 8.6%, and 12.2% amorphous contents in the carbon product obtained with NiZnFe_2_O_4_, NiMgFe_2_O_4_, and MgZnFe_2_O_4_, respectively. These findings suggest that the amorphous content can be reduced significantly by deliberately choosing the composite magnetic catalyst. One reason for having high purity in the solid product obtained with NiZnFe_2_O_4_ is the relatively defined particle shape and low agglomeration of the particles compared to the other catalysts. These results are consistent with the findings of SEM analysis. The amorphous content may include the unspent catalyst, amorphous carbon, and other impurities in the form of chemicals used during the fabrication of the plastic. Some char may also be present in the solid residue, which may convert to other forms during heating above 450°C.

The thermographs indicate that the onset temperature of product degradation is 380°C, with a peak temperature of 608°C.  Table 2 shows the initial and oxidation temperatures of all products. A similar behavior was observed in TGA curves in the case of all catalysts with slightly differing thermal decomposition temperatures. The graph shows that the samples exhibit good stability at temperatures below 350°C. No substantial weight loss was detected when the temperature was less than 380°C, as shown in Figure 8. At a temperature of ∼350°C, there was no sign of oxidation in any of the carbon samples [51]. A two-step weight loss mechanism can be seen in the composites. Initially, degradation occurs at a temperature between 382 and 410°C. The second degradation occurs between 620 and 632°C; thereafter, no change in weight loss is observed over temperature. This indicates that the temperature selected for pyrolysis of HDPE was appropriate for achieving maximum efficiency from the process [52].

### 3.4. Gas Analysis

**Figure 9:**
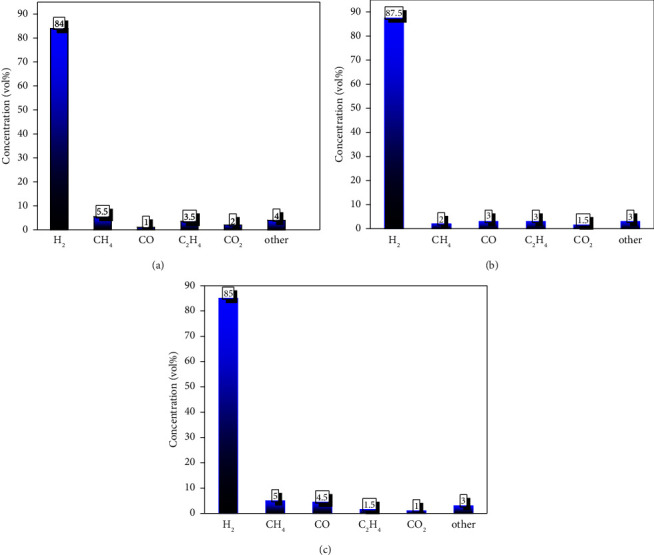
Gas compositions obtained with (a) NiZnFe_2_O_4_, (b) NiMgFe_2_O_4_, and (c) MgZnFe_2_O_4_ catalysts.

The pyrolytic gas composition differs from natural gas, as it contains alkenes, dienes, alkanes, and alkynes. Removing unsaturated hydrocarbons is essential for properly functioning gas turbines, gas engines, and fuel cells. The degradation of plastic waste produces noncondensable low-weight hydrocarbons (C_1_–C_5_). The gas formation depends on the process temperature and the type of catalyst used. The oxygenated compounds in the waste show that the obtained gases are primarily hydrocarbons ranging from C_1_–C_5_, H_2_, CO, to CO_2._ H_2_ is widely used as a feedstock in producing plastics, steel, pharmaceuticals, and chemicals [53]. Using H_2_ as green energy for vehicles will help reducing environmental pollution and climate change. Waste plastic can be used to produce hydrogen. Acomb et al. [54] used a fixed-bed reactor to obtain hydrogen gas from plastic pyrolysis. Figures 9(a)–9(c) report the volume percentage of H_2_, CH_3_, CO, and CO_2_ evolved from catalytic microwave pyrolysis of plastic using NiZnFe_2_O_4_, NiMgFe_2_O_4_, and MgZnFe_2_O_4_ catalysts.

The NiMgFe_2_O_4_ catalyst produced more hydrogen compared to other catalysts. This catalyst had a smaller particle size and relatively more agglomeration among the particles. The gas was composed of H_2_, CO, CH_4_, C_2_H_4_, and CO_2_ [55]. The hydrogen concentration of 84 vol%, 87 vol%, and 85 vol% was recorded with NiZnFe_2_O_4_, NiMgFe_2_O_4_, and MgZnFe_2_O_4_ catalysts, respectively. During the pyrolysis process, the maximal hydrogen evolved from HDPE was 127.9 mmol/g, 137.1 mmol/g, and 131.5 mmol/g, respectively [56]. The efficiency of H_2_ production was calculated by dividing the total mass of hydrogen present in all gas products by the theoretical mass of hydrogen in plastic [57].  Table 3 illustrates the gas composition (vol%) produced from microwave pyrolysis of HDPE with feedstock to catalyst ratio of 4 : 1 at 450°C. The produced gas mainly composed of a high value of H_2_, a small amount of CH_4_, CO, CO_2,_ C_2_H_4_, and other impurities.

### 3.5. FTIR Analysis of the Liquid Product

**Figure 10:**
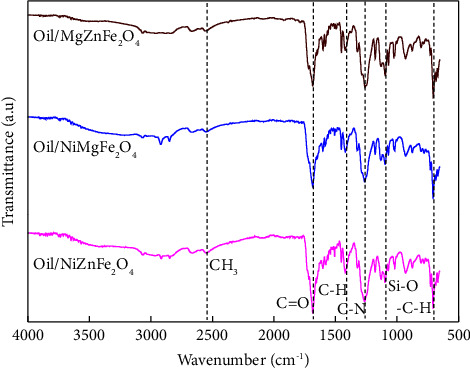
Functional groups of compounds observed in FTIR of liquid oil.

FTIR analysis was used to analyze the functional groups of HDPE pyrolytic oil. The oils were examined in the range of 500–4500 cm^−1^. The FTIR spectra of liquid oil produced from plastic waste are shown in Figure 10. The chemical compounds in the oil product are provided in Table 4. The spectrum indicates average hydrocarbon vibrations in the oil. No significant difference was observed in FTIR spectra of oils produced with NiZnFe_2_O_4_, NiMgFe_2_O_4_, and MgZnFe_2_O_4_ catalysts. Based on the observed absorption bands, the FTIR spectrum can be classified into 3100−2800 cm^−1^ range, 1700−1300 cm^−1^ range, and 1000−600 cm^−1^ range [58]. Alkane groups were observed between 2840 and 2640 cm^−1^, indicating the functional groups of alkenes CH_3_ stretching bands [59]. The highest absorption peak intensity was observed at 1687 cm^−1^, corresponding to the C=O stretching band of conjugated aldehydes. The aromatic amine's C-N stretching bending vibration was demonstrated by an intense peak at a frequency range of 1342−1260 cm^−1^ [60]. The band at 1090 cm^−1^ corresponds to Si-O stretching vibrations, while the aromatic ring -C-H was discovered at approximately 700 cm^−1^. The FTIR spectra of liquid oil extracted from HDPE employing ferrites as a catalyst revealed a high concentration of aliphatic hydrocarbons, such as alkanes and alkenes, but a low concentration of aromatic hydrocarbons such as aldehydes, ketones, and aromatic amines [61]. The FTIR spectra showed diesel, kerosine, and petrol range liquid fuel, and the highest intensity peaks showed the fingerprints of alkanes and alkenes. This liquid fuel can be processed further for widespread commercial use. NiZnFe_2_O_4_ catalysts produced more liquid oil than NiMgFe_2_O_4_ and MgZnFe_2_O_4_ catalysts.

### 3.6. Performance Comparison of Catalysts

**Figure 11:**
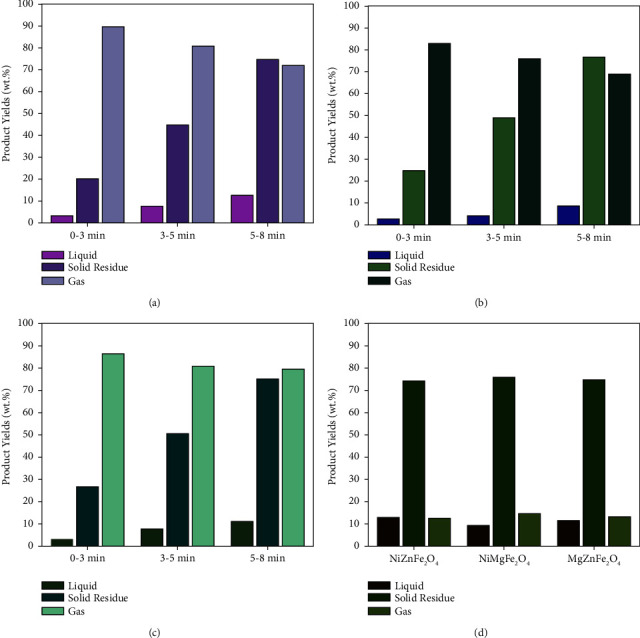
Time on stream evolution of products obtained with (a) NiZnFe_2_O_4_, (b) NiMgFe_2_O_4_, (c) MgZnFe_2_O_4,_ and (d) composition of product at the end of the process.

The heating temperature of the procedure was set at 450°C during microwave pyrolysis due to the low dielectric loss factor of the used magnetic catalysts. An appropriate ratio of catalyst to feedstock needs to be selected [62]. The used magnetic nanoparticles possessed both microwave absorption and catalytic characteristics [63]. A significant amount of hydrogen gas and carbon residue was obtained by using magnetic catalysts. These catalysts improved the capability of microwave energy transfer into heat energy. They enabled the pyrolysis temperature to rise more quickly, increasing the steam-reforming pyrolysis that produced H_2_ and CNTs. During pyrolysis, the organic materials rapidly crack at 450°C to produce mainly liquid oil [64]. These catalysts could rapidly raise the pyrolysis temperature and increase CNTs and H_2_ production. Compared with NiMgFe_2_O_4_, the solid yield and hydrogen gas with the NiZnFe_2_O_4_ and MgZnFe_2_O_4_ catalysts increased, while the liquid yield decreased to 9.1 wt% with NiMgFe_2_O_4_. The concentration of H_2_ produced by the pyrolysis process increased to 84% when NiMgFe_2_O_4_ served as the catalyst. Figures 11(a)–11(c) show the product yield, distribution, and composition over time. The H_2_ production remained 87.5%, 80.5% with NiZnFe_2_O_4_, and MgZnFe_2_O_4_ catalysts, respectively. The tested ferrite catalysts considerably enhance syngas yield and solid product compared to other magnetic ferrites with microwave absorption and catalyst properties due to good microwave absorption and catalytic properties [15]. NiZnFe_2_O_4_ produces liquid oil slightly more than other catalysts.  Figure 11(d) shows the product yield and distribution. The liquid oil was about 11 wt.%, 9 wt.%, and 8.5 wt.% using NiZnFe_2_O_4_, NiMgFe_2_O_4_, and MgZnFe_2_O_4_ catalysts, respectively. The gas evolved using these catalysts was about 12 wt.%, 12.5 wt.%, and 13 wt.%, respectively [65]. The gas mainly consisted of hydrogen and some other gases such as methane, carbon dioxide, and carbon monoxide. The liquid yield contained gasoline-range hydrocarbons.

The catalyst increases the speed of the reaction, converting waste plastic into useful products. The yields of end products are affected by the sample's residence time. Prolonged residence time increases the decomposition of the primary products, which causes the creation of thermally stable products, such as lightweight hydrocarbons or noncondensable gas [66]. The product distribution was split into 3 steps during pyrolysis. The yield of gas produced by the process increased from 80% to 90% in the first phase, solid residue increased from 15% to 22%, and just a small quantity of oil was found. The gas production reduced from 80% to 75% in the second step, but char residue increased from 35% to 40% and oil slightly increased. In comparison to prior stages of product yield evaluation, the oil and gas yields approached 13% and 14.5%, respectively, in the third step, while CNTs were boosted to 76.5%.  Table 5 depicts how various pyrolytic factors influenced the yield of liquid oil, gas, and solid.

A comparison of this study with the published literature is provided in Table 5. This comparison only included the studies conducted on catalytic pyrolysis of plastics at 450°C. This temperature is taken as an optimum condition in most of the published literature [67–69]. Rahman et al. [67] utilized Zeolite catalyst to produce 80.01% gas yield with a minimum solid yield of 3.2%. Our study was focused on the high yield of structured carbon residue. Babatabar et al. [69] used Ni-Cu/AC-Cao catalyst to produce 49.62% gas and 44% solid residue. Uthpalani et al. [11] used SiO_2_/Al_2_O_3_ catalyst to produce a high oil yield (74.05%) and a very low gas yield (5.08%). In our case, we produced a high yield of structured carbon residue (76.5%) using NiMgFe_2_O_4_ catalyst, a maximum oil yield of 11% using NiZnFe_2_O_4_ catalyst and a gas yield of 13% using MgZnFe_2_O_4_ catalyst. These three magnetic catalysts showed slight differences in oil, gas, and solid residue production.

## 4. Conclusions

We have offered a straightforward one-step microwave-initiated catalytic approach for rapidly transforming pulverized HDPE plastic waste into combustible gases and valuable carbon-based substances. NiZnFe_2_O_4_ catalyst had the least particle aggregation and large particle size. However, the shape and boundaries of NiZnFe_2_O_4_ catalyst particles were more defined than the other catalysts. This catalyst was recognized as a promising candidate for pyrolyzing into structure carbon. TGA analysis of the solid carbon residue showed 7.8%, 8.6%, and 12.2% amorphous contents in the carbon product produced using NiZnFe_2_O_4_, NiMgFe_2_O_4_, and MgZnFe_2_O_4_ catalysts, respectively. These findings suggest that the amorphous content can be reduced significantly by deliberately choosing the composite magnetic catalyst. Almost 87% of the H_2_ from HDPE was extracted in about 2–8 minutes. The H_2_ production was 137.1 mmol/g, and the H_2_ concentration in the produced gases reached 87.5 vol%. A high yield of structured carbon residue (76.5%) was produced using NiMgFe_2_O_4_ catalyst. The MgZnFe_2_O_4_ yielded the highest gas amount of 13%.

## Figures and Tables

**Table 1:** XRD analysis data of ferrite catalysts based on Figure 3.

Samples	Lattice parameters (A)	Strains	Grain size (nm)
Ni_0.5_Zn_0.5_Fe_2_O_4_	8.4071	0.002166	17
Ni_0.5_Mg_0.5_Fe_2_O_4_	8.4100	0.002595	39
Mg_0.5_Zn_0.5_F_2_O_4_	8.4059	0.002387	35.46

**Table 2:** Summary of TGA analysis of CNTs.

Catalyst types	Initial temperature (°C)	Oxidation temperature (°C)
NiZnFe_2_O_4_	382	620
NiMgFe_2_O_4_	384	624
MgZnF_2_O_4_	385	632

**Table 3:** Composition of gas evolved during the pyrolysis of HDPE using different magnetic catalysts.

Gases	NiZnFe_2_O_4_	NiMgFe_2_O_4_	MgZnFe2O4
	Gas composition (vol.%)		
H_2_	84.0	87.5	85
CH_4_	6.5	5	7
CO	3.5	3.5	5.5
C_2_H_4_	3	3	1.5
CO_2_	1.5	1.5	1
Others	4	3	3

**Table 4:** Summary of FTIR analysis of oil product.

Wavelength ranges (cm^−1^)	Functional groups	Compound types
2840−2640	CH_3_ stretching band	Alkanes
1710−1680	C=O stretching band	Conjugated aldehydes/ketones
1683−1410	C-H stretching bend	Alkenes
1342−1260	C-N stretching band	Aromatic amine
1200−1090	Si-O stretching bend	Alkenes
800−700	-C-H stretching bands	Alkenes

**Table 5:** A comparison of catalyst effect on the product yield from pyrolysis of plastics at 450°C.

Catalysts	Pyrolysis oil	Solid yield	Gas yield	References
Zeolite	16.7%	3.2%	80.01%	[67]
Na/ZSM-5	56%	20%	24%	[68]
Ni-Cu/AC-Cao	10.04%	44%	49.62%	[69]
Activated alumina	21.08%	16.01%	62.01%	[70]
Fe_2_O_3_/Al_2_O_3_	12%	18%	70%	[71]
Fe/Ni/Mg	26.43%	27.17%	44.36%	[72]
SiO_2_/Al_2_O_3_	74.05%	19.07%	5.08%	[11]
MgZnFe_2_O_4_	8.5	75.5	13	Current study
NiMgFe_2_O_4_	9	76.5	12.5	Current study
NiZnFe_2_O_4_	11	75	12	Current study

## Data Availability

The data used to support the findings of this study are included within the article.
